# Genetic polymorphisms in *Plasmodium falciparum* chloroquine resistance genes, *pfcrt* and *pfmdr1*, in North Sulawesi, Indonesia

**DOI:** 10.1186/s13104-017-2468-1

**Published:** 2017-04-04

**Authors:** Patrick Reteng, Visia Vrisca, Inka Sukarno, Ilham Habib Djarkoni, Jane Angela Kalangi, George Eduardo Jacobs, Lucky Ronald Runtuwene, Yuki Eshita, Ryuichiro Maeda, Yutaka Suzuki, Arthur Elia Mongan, Sarah Maria Warouw, Junya Yamagishi, Josef Tuda

**Affiliations:** 1 Department of Medicine, Sam Ratulangi University, Kampus Unsrat, Bahu, Manado, 95115 Indonesia; 2 Department of Medical Genome Sciences, University of Tokyo, Kashiwa, Chiba 277-8562 Japan; 3 Faculty of Medicine, Oita University, Yufu, Oita 879-5593 Japan; 4 Faculty of Tropical Medicine, Mahidol University, Bangkok, 10400 Thailand; 5 Research Center for Zoonosis Control, Hokkaido University, North 20, West 10 Kita-ku, Sapporo, Hokkaido 001-0020 Japan; 6 Department of Human Sciences, Obihiro University of Agriculture and Veterinary Medicine, Obihiro, Hokkaido 080-8555 Japan; 7 Global Station for Zoonosis Control, GI-CoRE, Hokkaido University, North 20, West 10 Kita-ku, Sapporo, Hokkaido 001-0020 Japan

**Keywords:** Chloroquine, Genetic polymorphism, Indonesia, Malaria, Multiplex sequencing, North Sulawesi, PCR–RFLP, *pfcrt*, *pfmdr1*, *Plasmodium falciparum*

## Abstract

**Background:**

Malaria still poses one of the major threats to human health. Development of effective antimalarial drugs has decreased this threat; however, the emergence of drug-resistant *Plasmodium falciparum*, a cause of Malaria, is disconcerting. The antimalarial drug chloroquine has been effectively used, but resistant parasites have spread worldwide. Interestingly, the withdrawal of the drug reportedly leads to an increased population of susceptible parasites in some cases. We examined the prevalence of genomic polymorphisms in a malaria parasite *P. falciparum*, associated with resistance to an antimalarial drug chloroquine, after the withdrawal of the drug from Indonesia.

**Results:**

Blood samples were collected from 95 malaria patients in North Sulawesi, Indonesia, in 2010. Parasite DNA was extracted and analyzed by polymerase chain reaction–restriction fragment length polymorphism (PCR–RFLP) for *pfcrt* and *pfmdr1*. In parallel, multiplex amplicon sequencing for the same genes was carried out with Illumina MiSeq. Of the 59 cases diagnosed as *P. falciparum* infection by microscopy, PCR–RFLP analysis clearly identified the genotype 76T in *pfcrt* in 44 cases. Sequencing analysis validated the identified genotypes in the 44 cases and demonstrated that the haplotype in the surrounding genomic region was exclusively SVMNT. Results of *pfmdr1* were successfully obtained for 51 samples, where the genotyping results obtained by the two methods were completely consistent. In *pfmdr1*, the 86Y mutant genotype was observed in 45 cases (88.2%).

**Conclusions:**

Our results suggest that the prevalence of the mutated genotypes remained dominant even 6 years after the withdrawal of chloroquine from this region. Diversified haplotype of the resistance-related locus, potentially involved in fitness costs, unauthorized usage of chloroquine, and/or a short post-withdrawal period may account for the observed high persistence of prevalence.

**Electronic supplementary material:**

The online version of this article (doi:10.1186/s13104-017-2468-1) contains supplementary material, which is available to authorized users.

## Background

Malaria caused by *Plasmodium falciparum* remains a major health concern, particularly in tropical and subtropical regions. *P. falciparum* uses anopheline mosquitoes as a vector and spreads rapidly in the tropics and subtropics, where 3.3 billion people are at risk of contracting the parasite [1]. According to a 2016 World Health Organization report, >212 million cases of malaria were reported annually and approximately 429,000 people died from malaria worldwide [1]. In Southeast Asia, 15 million cases of malaria (7% global cases) and 26,000 deaths (6% global deaths) are reportedly associated with malaria each year [1].

Malaria is curable and preventable; however, the disease has not yet been completely eradicated. *P. falciparum* develops resistance against available medication, which explains the failed eradication of malaria; medication plays an important role in malaria control programs [2, 3]. Reports suggest that *P. falciparum* has now developed resistance to most antimalarial drugs, including chloroquine and its derivatives, sulfadoxine–pyrimethamine, mefloquine, and artemisinin [2–6]. Indeed, chloroquine was the standard antimalarial drug; however, chloroquine-resistant *P. falciparum* emerged in the late 1950s and spread worldwide [2]. It is widely accepted that several polymorphisms play important roles in chloroquine-resistant *P. falciparum*, particularly a threonine substitution at codon 76 in the *P. falciparum* chloroquine-resistant transporter (*pfcrt*) and a tyrosine substitution at codon 86 in *P. falciparum* multidrug-resistant protein (*pfmdr1*) [7–11].

Chloroquine resistance is widely distributed; however, reports from some African countries indicate a decline in the resistant parasite population after chloroquine discontinuation [12–16], although this reduction in resistance varied between countries. A study in Malawi demonstrated a marked decrease in the prevalence of the chloroquine-resistant marker from 85 to 15% 13 years after chloroquine discontinuation [16], where its incidence was only 1/685 in 2009 [17]. Similar observations have been reported from other African countries [12, 14]. However, 76T remains highly prevalent in Brazil [18], the Thai–Myanmar border [19], and Pakistan [20].

**Fig. 1:**
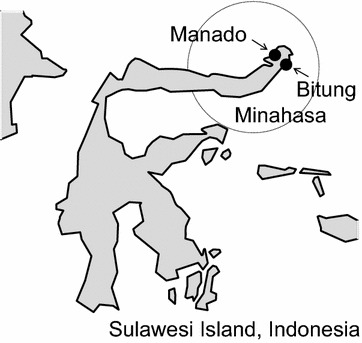
Geographical representation of the sampling sites. *Dots* represent cities where blood samples were collected. The *dashed circle* represents the Minahasa region where a previous study was conducted [21]

North Sulawesi is one of the malaria-endemic regions of Indonesia. A previous study detected a high prevalence of both 76T and 86Y polymorphisms (94 and 95%, respectively) among isolates from Minahasa, North Sulawesi (Fig. 1) [21]. The Indonesian government had to change its malaria treatment policy because of the high rate of chloroquine resistance, and the administration of artemisinin-based combination therapies began in 2004. Subsequently, dihydroartemisinin piperaquine became the dominant therapy [3]. As mentioned above, chloroquine-sensitive *P. falciparum* returned to some regions after the discontinuation of chloroquine. If the use of chloroquine can be reconsidered, its benefits would be considerable because chloroquine is a cost-effective drug without known severe side-effects. Most importantly, the reuse of pre-existing drugs would conserve the limited repertoire of antimalarial drugs. To better understand the changes in the resistant genotypes, we determined the prevalence of polymorphisms in *pfcrt* and *pfmdr*1 after chloroquine discontinuation in North Sulawesi, Indonesia.

## Methods

### Sample collection

Blood samples were collected from 95 malaria patients clinically diagnosed at hospitals in Manado and Bitung, North Sulawesi, Indonesia (Fig. 1), from August to December 2010. The collected blood samples were diagnosed via Giemsa staining and microscopic analysis by medical staff members at each hospital and collected on FTA Elute cards (GE Healthcare Life Sciences, Little Chalfont, UK).

### Polymerase chain reaction–restriction fragment length polymorphism (PCR–RFLP) analysis

Parasite DNA was extracted from dried filter blood spots by boiling them at 95 °C for 15 min. PCR–RFLP analysis of *pfcrt* and *pfmdr1* was conducted as described previously [22]. In brief, PCR was conducted using KAPA 5× buffer (containing 7.5 mM MgCl_2_), 2.5 mM MgCl_2_, 0.25 mM each dNTP, 0.5 μM forward and reverse primers (Table 1), 0.625 U Taq, and 5 μL of extracted DNA in a total volume of 20 μL. For the nested PCR analyses, 2 μL of the 100× diluted PCR product from the first amplification step was used as a template in the second step. Restriction enzyme digestion was performed with 7 μL (*pfcrt*) or 5 μL (*pfmdr1*) of the PCR products and two units of *Apo*I for *pfcrt* or *Afl*III for *pfmdr1*. A laboratory clone (3D7) was also amplified and digested along with the samples, which served as a positive control. The digested products were stained with ethidium bromide and separated on a 2% agarose gel.

**Table 1:** Primers used in the PCR–RFLP analyses

*Pfcrt*	
1st amplification	
*pfcrt*76F1st	
GCGCGCGCATGGCTCACGTTTAGGTGGAG	
*pfcrt*76R1st	
GGGCCCGGCGGATGTTACAAAACTATAGTTACC	
2nd amplification for PCR–RFLP	
*pfcrt*76F2nd	
TGTGCTCATGTGTTTAAACTT	
*pfcrt*76R2nd	
CAAAACTATAGTTACCAATTTTG	
2nd amplification for NGS amplicon sequencing	
*pfcrt*76F2ndNGStag	
ACACTCTTTCCCTACACGACGCTCTTCCGATCTNNTGTGCTCATGTGTTTAAACTT	
*pfcrt*76R2ndNGStag	
GTGACTGGAGTTCAGACGTGTGCTCTTCCGATCTNNCAAAACTATAGTTACCAATTTTG	
*pfmdr*1	
1st amplification	
*pfmdr*86F	
GCGCGCGTTGAACAAAAAGAGTACCGCTG	
*pfmdr*86R	
GGGCCCTCGTACCAATTCCTGAACTCAC	
2nd amplification for PCR–RFLP	
*pfmdr*86F	
TTTACCGTTTAAATGTTTACCTGC	
*pfmdr*86R	
CCATCTTGATAAAAAACACTTCTT	
2nd amplification for NGS amplicon sequencing	
*pfmdr*86F2ndNGStag	
ACACTCTTTCCCTACACGACGCTCTTCCGATCTNNACCGTTTAAATGTTTACCTGC	
*pfmdr*86R2ndNGStag	
GTGACTGGAGTTCAGACGTGTGCTCTTCCGATCTNNCATCTTGATAAAAAACACTTCTT	

### Multiplex amplicon sequencing with MiSeq and genotyping

The primary PCR reaction was performed as described above. The second nested PCR analyses used 2 μL of the 100× diluted PCR product from the first amplification step as template, with added tab nucleotides (Table 1). For the third PCR, with added dual indices, 2 μL of the 100× diluted PCR product from the second amplification step and TruSeq^®^ DNA HT and RNA HT Sample Prep Kits (Illumina) were used. The amplicons were quantified with an Agilent 2200 TapeStation (Agilent), and then mixed to achieve the same molecular number. The samples were subjected to MiSeq (Illumina), and then sequenced with a MiSeq v3 Reagent Kit v3 (600-cycle). The short read sequences obtained were mapped onto the *pfcrt* and *pfmdr1* coding sequences extracted from *P. falciparum* genome version 13, which was obtained from plasmoDB [23] by bowtie2 [24]. To identify genetic polymorphisms, the mapped results were analyzed with GATK [25].

### Statistical tests

Regional differences in the genotypes were examined with Fisher’s exact test.

## Results and discussion

### Detection of the frequencies of the mutant genotypes of *pfcrt* and *pfmdr1*

**Fig. 2:**
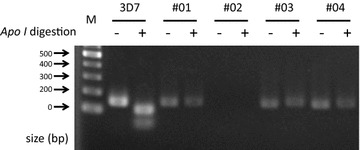
Representative images of the PCR–RFLP results. *M*:100-bp marker, *3D7* Control sample prepared using DNA extracted from the chloroquine-susceptible 3D7 strain

**Fig. 3:**
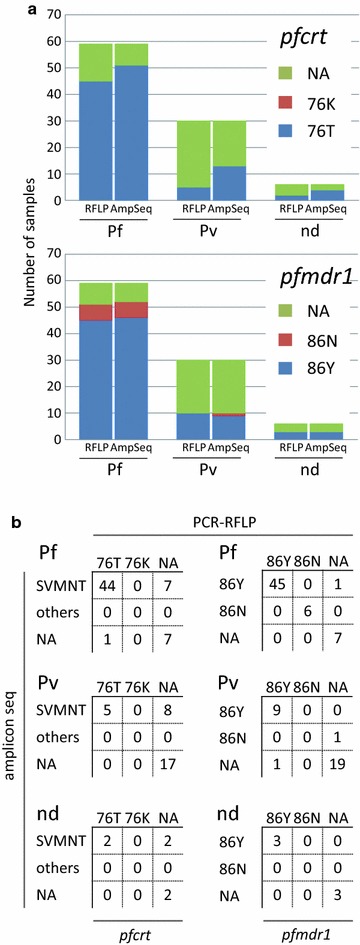
Summary of the diagnosis and genotyping by PCR–RFLP, and the amplicon sequences. **a** The *upper and lower panels* show the results for *pfcrt* and *pfmdr1*, respectively. As shown by the *two bars on the left*, 59 samples were diagnosed as *P. falciparum* or *P. falciparum*–*P. vivax*-mixed infections according to Giemsa tests conducted by regional doctors. As shown by the *two bars in the middle*, 30 were diagnosed as *P. vivax* infections. As shown by the *two bars on the right*, six had no medical descriptions. In each set of bars, the *left bar* represents the genotype determined by PCR–RFLP and the *right bar* represents that detected by amplicon sequencing. **b** Cross tables comparing the genotyping results obtained by amplicon sequencing and PCR–RFLP. The *tables on the left and right* show the results for *pfcrt* and *pfmdr1*, respectively. *NA* not available because of a lack of PCR amplicons, *nd* no medical descriptions available from the collected sample

**Fig. 4:**
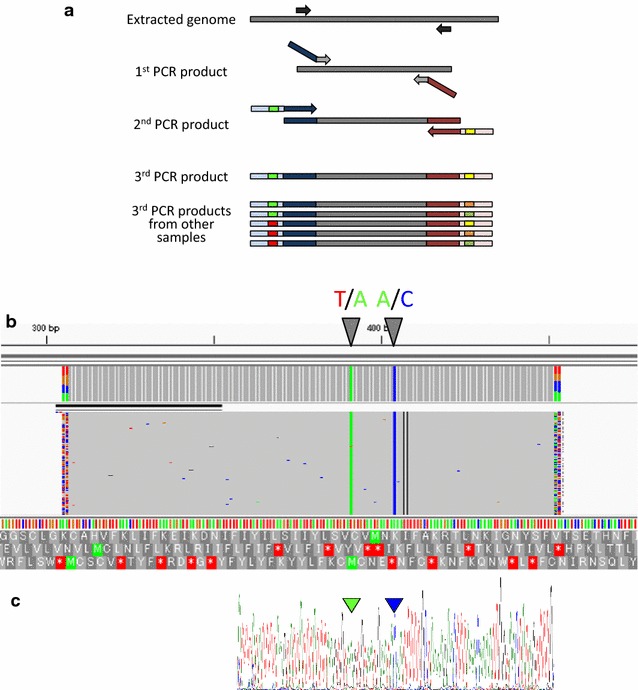
Scheme of next generation-based high-throughput multiplex amplicon sequencing and a representative result. **a** Target regions amplified by nested PCR. In the second PCR, primers with tags for the third PCR were used. In the third PCR, the primers had unique pairs of indices for multiplex sequencing. After sequencing with MiSeq, the reads obtained were separated according to the indices. *Red* and *blue lines* represent index sequences with priming targets for Illumina sequencing. **b** Snapshot of the mapped reads for sample ID A1 with IGV. *Arrowheads* represent the called variants. The reference is the sequence for the 3D7 strain. **c** Validation by Sanger sequencing of the corresponding region. *Arrowheads* represent polymorphisms detected by sequencing

In this study, we collected 95 blood samples that had been clinically diagnosed as malaria cases by local doctors. Among these samples, 59 were diagnosed by Giemsa staining as *P. falciparum* infections or possibly mixed infections of *P. falciparum* and *P. vivax*. Thirty cases were infections with *P. vivax*. No medical evidence was recorded for the remaining 6 cases. Using these samples, we conducted PCR–RFLP assays of the 227th base in *pfcrt* to identify the drug resistance-related genotypes at amino acid 76 (Fig. 2). We obtained clear results from 45/59 cases, which were initially diagnosed as *P. falciparum* infections (Fig. 3; Additional file 1: Table S1). In these 45 cases (100%), we identified the genotype “C,” which encodes 76T, and thus, a chloroquine-resistant *pfcrt*. We conducted similar analyses for the 36 remaining non-*P. falciparum* cases. We found that the *P. falciparum* genome was detected in five cases diagnosed as *P. vivax* infections and two cases without any medical records, possibly due to their ambiguous diagnoses. The identified genotype also encoded a 76T in these cases. In addition, to validate the correct identifications of the genotypes, we subjected the amplified PCR products to multiplex sequencing on the Illumina MiSeq platform (Fig. 4a, b). We examined all 45 cases and confirmed that the genotypes of the *P. falciparum* infections were 76T (see “Methods” for details of the single-nucleotide polymorphism procedure), except for one where we failed to obtain an amplicon by PCR (Fig. 3; Additional file 1: Table S1). We validated the sequences using the Sanger method for four randomly selected samples, which confirmed that they were identical (Fig. 4c). In addition to validating the sequences, multiplex amplicon sequencing allowed us to identify the exact genotypes in the surrounding region, i.e., the haplotypes. For amino acids 72–76, we found that all cases had the SVMNT haplotype and no case was identified with the CVIET haplotype. Non-synonymous variations at codon 72S were identified as “agt” and “tct” in 41 and 3 specimens, respectively (Additional file 1: Table S1).

We conducted PCR–RFLP assays of the *pfmdr1* gene in parallel (Fig. 3; Additional file 1: Table S1). We obtained clear results from 51/59 samples. We also detected *P. falciparum* genomes in 10 cases initially diagnosed as *P. vivax* and 3 cases without any medical records. Among the 51 samples, the 86Y and 86N genotypes were identified in 45 (88.2%) and 6 (11.8%) cases, respectively. We conducted amplicon sequencing of these samples on MiSeq, which confirmed the correct genotype results in all cases. Based on these results, we can make the following conclusions. First, conventional Giemsa staining is occasionally imprecise and further confirmation is needed to precisely detect the infecting parasite species. Second, PCR–RFLP is a convenient and accurate method, although further sequencing analysis can provide extensive information associated with the surrounding bases. Finally, and most importantly, the dominant population of *P. falciparum* in this area still has a chloroquine-resistant genotype. We also collected and analyzed blood samples from two neighboring regions, i.e., Manado and Bitun, in North Sulawesi, but found no significant differences between the areas in terms of the prevalence of 76T and 86Y mutants (*p* = 1.000 and 0.218, respectively).

### Comparison of the observed prevalence of *pfcrt* genotype with that in previous studies

Several previous studies have investigated the prevalence of the mutant genotypes of *P. falciparum*. A study that covered all of Indonesia in 2004 based on PCR–RLFP analyses showed that most of the genotypes in North Sulawesi were 76T [21]. However, compared to previous studies, we found a slight increase in the prevalence of 76T mutation (from 94.4 to 100%), despite the lack of clear statistical significance (*p* = 0.290).

Another previous study showed that in Lombok, Indonesia, codons 72–76 comprised SVMNT in most cases (87.5%) and only a few isolates possessed the Southeast Asia type of CVIET (10.4%) during 2002 [26]. The high prevalence of 76T was a common feature of all our observations, but we observed no cases of the CVIET haplotype in North Sulawesi. It is known that SVMNT is dominant in the Pacific region, such as Indonesia, Papua New Guinea, and the Philippines in Southeast Asia. By contrast, CVIET is dominant in Thailand, Vietnam, and Myanmar [27, 28], and the CVIET haplotype is assumed to have been spread from there to Africa [29]. Therefore, our finding that SVMNT occurred exclusively in North Sulawesi may reflect the distinct geological origins of the drug-resistant genotypes to some extent.

Intriguingly, the results of our study were very different from those obtained in previous studies conducted in Malawi and other African countries. In Malawi, the prevalence of the *pfcrt* 76T genotype decreased significantly from 85% in 1992 to 13% in 2000 [16, 30]. Subsequently, it was only present in 1/685 (0.1%) cases in 2009 [17]. In agreement, a field study showed that the susceptibility rate of the parasites to chloroquine increased from 50% in 1993 to 99% in 2005 [30]. Similar reductions in the prevalence of the *pfcrt* 76T genotype have also been reported from Kenya, Senegal, and other countries that are mainly located in Africa, although their remission rates were less drastic than that in Malawi [12, 14, 31]. Based on these studies, it is suggested that the mutants are fitter than the wild type under the selective pressure of chloroquine. In contrast, the advantage should disappear without the drug. Thus, the corresponding mutation genotypes may have disappeared after a certain time following the drug’s withdrawal.

To rationalize the observed reduction, it should also be hypothesized that the mutants are less fit than the wild type without the drug pressure. It seems to be true in CVIET, which is a major mutated haplotype around the 76T genotype and observed in the regions where the reduction of 76T was reported. In contrast, the SVMNT haplotype, which we found was dominant in North Sulawesi, is likely to be no less fit than the wild type [29]. Indeed, the fixed prevalence of 76T was also reported even after chloroquine withdrawal in Venezuela, where the SVMNT haplotype is similarly dominant [32]. Thus, differences in the biological features of the CVIET and SVMNT haplotypes with respect to their fitness may contribute to the distinct variations in their prevalence. Another even more confusing factor is the administration policy for the use of antimalarial drugs, where incomplete drug withdrawal and unauthorized chloroquine usage may have exposed the parasite to subtherapeutic concentrations, thereby contributing to the fixed mutant genotype. It has been shown that SVMNT is also involved in resistance to amodiaquine, which is used in a combination of artesunate–amodiaquine (AA) [27]. In Indonesia, AA is used despite the current policy recommending dihydroartemisinin piperaquine (DHP). Therefore, suboptimal administration of AA is potentially involved in the sustained SVMNT haplotype, and the inverse effect by strict substitution from AA to DHP may be expected. In addition, misdiagnosis may contribute to the confusion. Hence, further biological studies combined closely with epidemiological and possibly social science studies may yield a more thorough understanding of the prevalence dynamics of the mutant genotypes and their controls.

### Prevalence of the genotypes of *pfmdr1*

It is known that the *pfmdr1* 86Y genotype is widespread throughout Indonesia (Table 2) [21]. The reported prevalence rates are 15.8 and 26.7% in Flores and East Nusa Tenggara (the eastern part of Indonesia) and in Armopa (West Papua), respectively [21]. By contrast, in western Indonesia, the reported prevalence of 86Y polymorphism is 100% [21]. Sulawesi, located in the middle of these regions, reported the prevalence rates for *pfmdr*1 86Y polymorphism as 95.0% in Minahasa, North Sulawesi, and 62.5% in Mamuju, South Sulawesi. The prevalence rate detected in the present study was between these two values (88.2%; *p* = 0.664 and 0.029, respectively). For the Asian countries surrounding Indonesia, the reported prevalence rates are similar to those in western Indonesia or Sulawesi, as well as cases from the most southern part of Thailand (96.3%; *p* = 0.018, compared with our results). However, the reported prevalence rates in Peninsular Malaysia (5.3%, *p* = 0.000), Cambodia (2.15%, *p* = 0.000), western Thailand (3.1%, *p* = 0.000), and the upper southern part of Thailand (36.4%, *p* = 0.000) differed from ours [19, 33–36]. Other studies have described almost equal prevalence rates for pfmdr1 86Y in African countries such as Kenya (81.6%, *p* = 0.545) or even lower prevalence in Benin (57.1%, *p* = 0.000), Malawi (22.7%, *p* = 0.000), and Senegal (14.9%, *p* = 0.000) [13, 14, 30, 37]. Note that one of the lowest prevalence rates for the mutant genotype 86Y was reported from Malawi, where the prevalence of the mutant *pfcrt* also decreased dramatically [16, 30]. However, as discussed in these studies, the geographical distribution of 86Y polymorphism is complicated and does not always coincide with geographical distribution of *pfcrt* polymorphism. Furthermore, unlike the mutation at 76T in *pfcrt*, a clear reduction in the mutant genotype has not yet been reported after the withdrawal of chloroquine. Indeed, it is known that polymorphisms in *pfmdr*1 are involved in resistance to not only chloroquine but also amodiaquine, mefloquine, and lumefantrine [9, 37–39]. In this study, a small trend of reduction in the six years (95.0 to 88.2%) was observed; however, it was not statistically significant (*p* = 0.664). Besides, the regions we investigated were not similar enough for the difference to be significant. Therefore, continuous and robust monitoring should be considered to determine the effect of chloroquine withdrawal on the prevalence of 86Y genotype in this region.

**Table 2:** Prevalence rates of *pfcrt* 76T and *pfmdr1* 86Y polymorphisms in this and previous studies

Year	Region	*pfcrt*, 76T	*pfmdr1*, 86Y	Reference
2010	Manado	26/26 (100%)	24/29 (82.8%)	This study
2010	Bitung	18/18 (100%)	21/22 (95.5%)	This study
2010	(Total)	44/44 (100%)	45/51 (88.2%)	This study
2004	Flores (East NusaTenggara)	20/20 (100%)	3/19 (15.8%)	[21]
2004	Armopa (West Papua)	13/15 (86.7%)	4/15 (26.7%)	[21]
2004	Minahasa (North Sulawesi)	17/18 (94.4%)	19/20 (95.0%)	[21]
2004	Mamuju (South Sulawesi)	16/16 (100%)	10/16 (62.5%)	[21]
2004	Nias (North Sumatra)	20/20 (100%)	19/19 (100%)	[21]
2004	Hanura (Lampung)	25/25 (100%)	41/41 (100%)	[21]
2004	Kokap (Central Java)	21/21 (100%)	28/28 (100%)	[21]
2004	Kutai (East Kalimantan)	19/19 (100%)	28/28 (100%)	[21]
2010	Peninsular Malaysia	39/75 (52.0%)	4/75 (5.3%)	[33]
2009	Upper southern part of Thailand	66/66 (100%)	24/66 (36.4%)	[35]
2009	Lower southern part of Thailand	492/492 (100%)	474/492 (96.3%)	[35]
2004	Cambodia	NA*	2/93 (2.2%)	[36]
2001	West part if Thailand	271/271 (100%)	8/270 (3.0%)	[34]
2006	Kenya	30/48 (62.5%)	31/38 (81.6%)	[14]
2011	Benin	200/213 (93.9%)	121/212 (57.1%)	[13]
2000	Malawi	10/75 (13.3%)	10/44 (23.0%)	[30]
2009	Senegal	NA*	26/174 (14.9%)	[37]

## Conclusion

In this study, using samples obtained from malaria patients from North Sulawesi, Indonesia, we examined the chloroquine resistance polymorphisms in *P. falciparum*, i.e., 76T in *pfcrt* and 86Y in *pfmdr1*, by PCR–RFLP followed by multiplex amplicon sequencing. We emphasized the power of employing the multiplex amplicon sequencing method as well as confirming the convenience of using the conventional RFLP–PCR method. The cost of sequencing is decreasing rapidly; thus, a more comprehensive overview of the changes in genotypes throughout the world can be obtained by targeted sequencing of a larger number of genes in parasites or even whole genome sequencing. The results obtained in the present study showed that the prevalence rates of the mutant genotypes were 100 and 88.2% for 76T in *pfcrt* and 86Y in *pfmdr1*, respectively. We consider that the fixation of 76T mutations can be explained by the incomplete withdrawal of the drug, the short time interval since the withdrawal of chloroquine, and/or the possible equal fitness costs of the susceptible and resistant SMVNT genotypes. This suggests that re-emergence of the use of chloroquine is by no means guaranteed and that national health authorities should consider the results of continuous molecular surveillance when formulating their malaria treatment policies. To obtain definitive conclusion and rationalize these assumptions, the size of our cohort is not comprehensive enough and more detailed epidemiological information might be required. The multiplex amplicon sequencing system we applied is, however, supportive to this end.

## Additional file

**Additional file 1: Table S1.** Table of the observed genotypes in this study.

## Abbreviations

*P. falciparum*: *Plasmodium falciparum*
*P. vivax*: *Plasmodium vivax*
PCR–RFLP: polymerase chain reaction–restriction fragment length polymorphism
*pfcrt*: *P. falciparum* chloroquine-resistant transporter
*pfmdr1*: *P. falciparum* multidrug-resistant protein
AA: artesunate–amodiaquine
DHP: dihydroartemisinin piperaquine
